# Supplementary material for: Inulin-Based Polymeric Micelles Functionalized with Ocular Permeation Enhancers: Improvement of Dexamethasone Permeation/Penetration through Bovine Corneas
Source: Pharmaceutics. 2021 Sep 9;13(9):1431. doi: 10.3390/pharmaceutics13091431 (PMC8472490; doi:10.3390/pharmaceutics13091431)
Supplement: Supplementary file 1 [file pharmaceutics-13-01431-s001.zip › pharmaceutics-1331897-supplementary.pdf]

pharmaceutics

MDPI

Supplementary Materials: Inulin-Based Polymeric Micelles Functionalized with Ocular Permeation Enhancers: Improvement of Dexamethasone Permeation/Penetration through Bovine Corneas

Giulia Di Prima, Mariano Licciardi *, Flavia Bongiovì, Giovanna Pitarresi and Gaetano Giammona

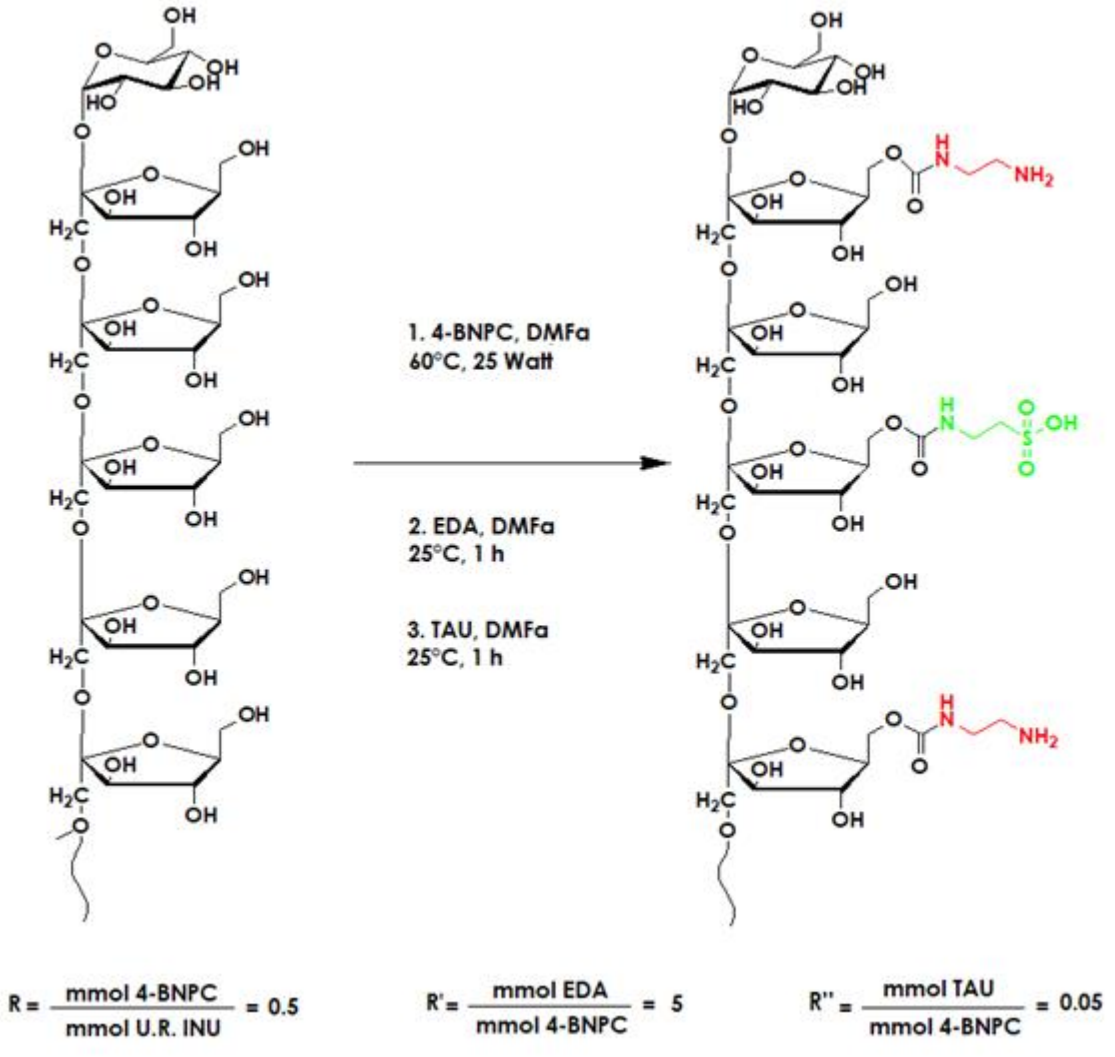

Scheme S1. Synthesis of INU-EDA-TAU.

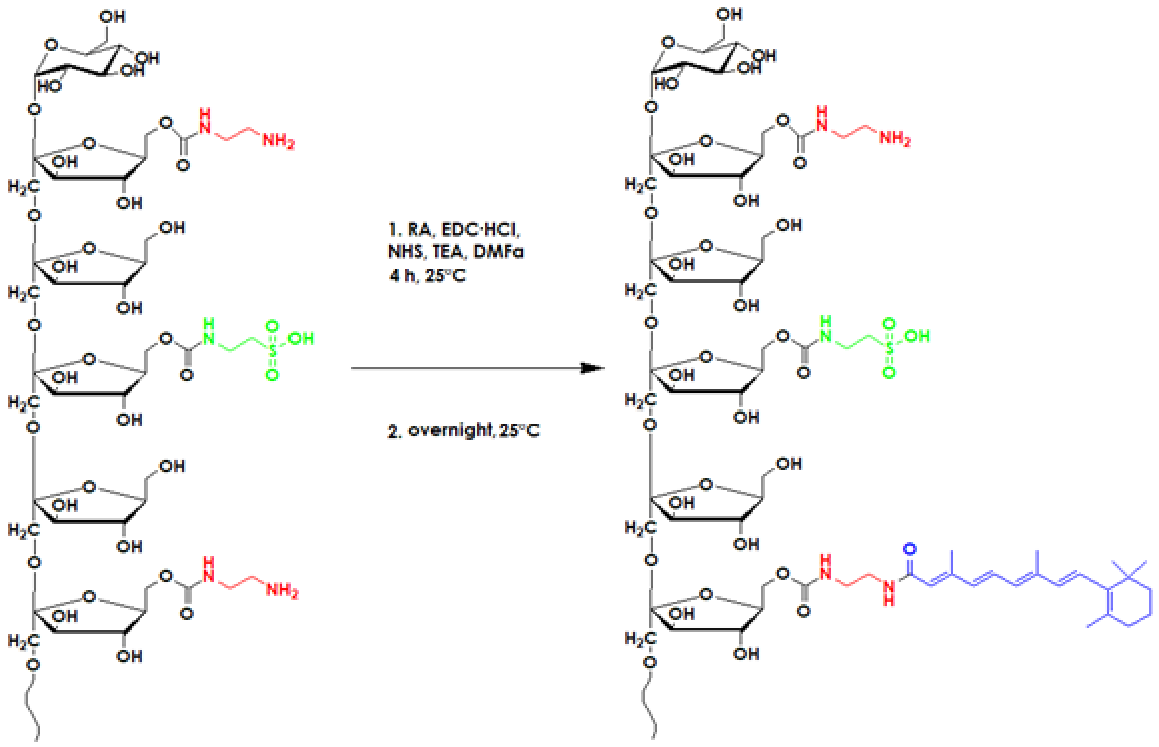

$$R = \frac{\text{mmol RA}}{\text{mmol U.R. INU-EDA}} = 0.15 \qquad R' = \frac{\text{mmol EDC·HCl}}{\text{mmol RA}} = 1.5 \qquad R'' = \frac{\text{mmol NHS}}{\text{mmol RA}} = 1.5$$

Scheme S2. Synthesis of INU-EDA-TAU-RA.

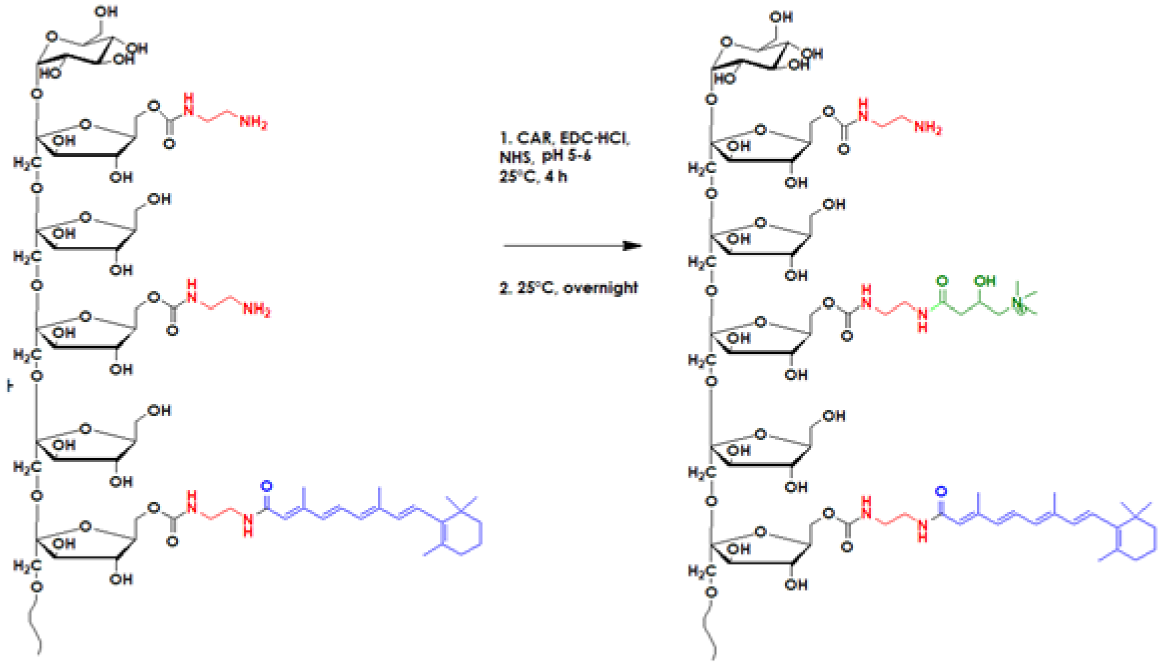

$$R = \frac{\text{mmol CAR}}{\text{mmol U.R. INU-EDA-RA}} = 0.07 \qquad R' = \frac{\text{mmol EDC·HCl}}{\text{mmol CAR}} = 1.5 \qquad R'' = \frac{\text{mmol NHS}}{\text{mmol CAR}} = 1.5$$

Scheme S3. Synthesis of INU-EDA-RA-CAR.

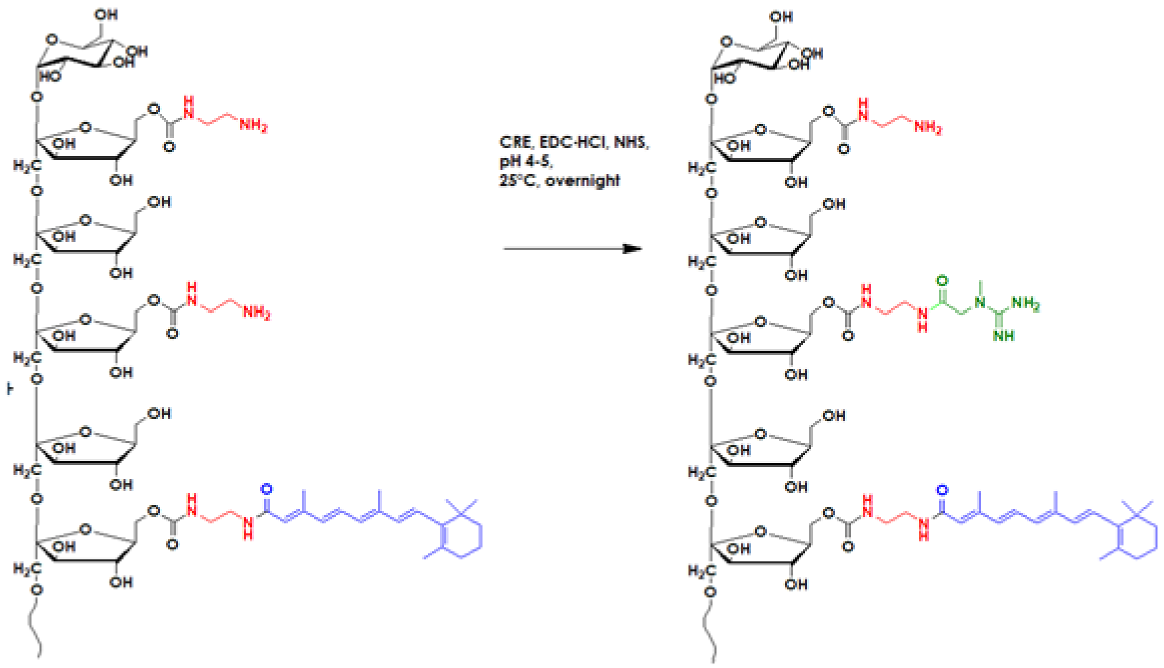

$$R = \frac{\text{mmol CRE}}{\text{mmol U.R. INU-EDA-RA}} = 0.03 \qquad R' = \frac{\text{mmol EDC·HCl}}{\text{mmol CRE}} = 1.5 \qquad R'' = \frac{\text{mmol NHS}}{\text{mmol CRE}} = 1.5$$

Scheme S4. Synthesis of INU-EDA-RA-CRE.

A)

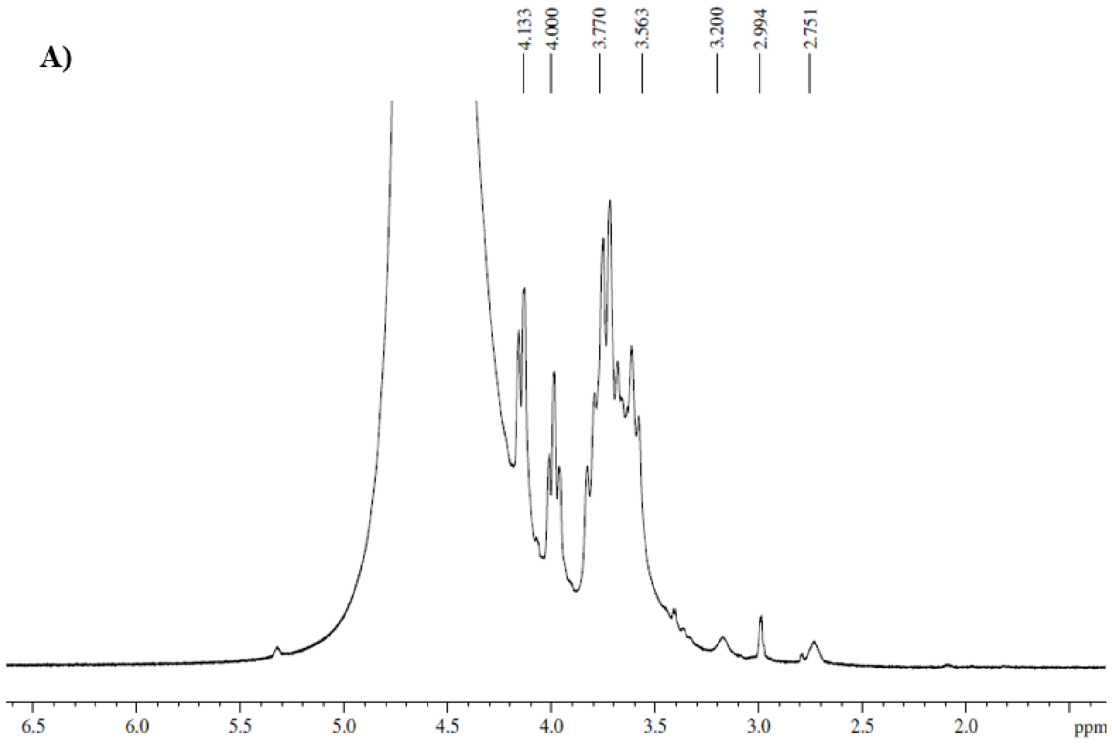

B)

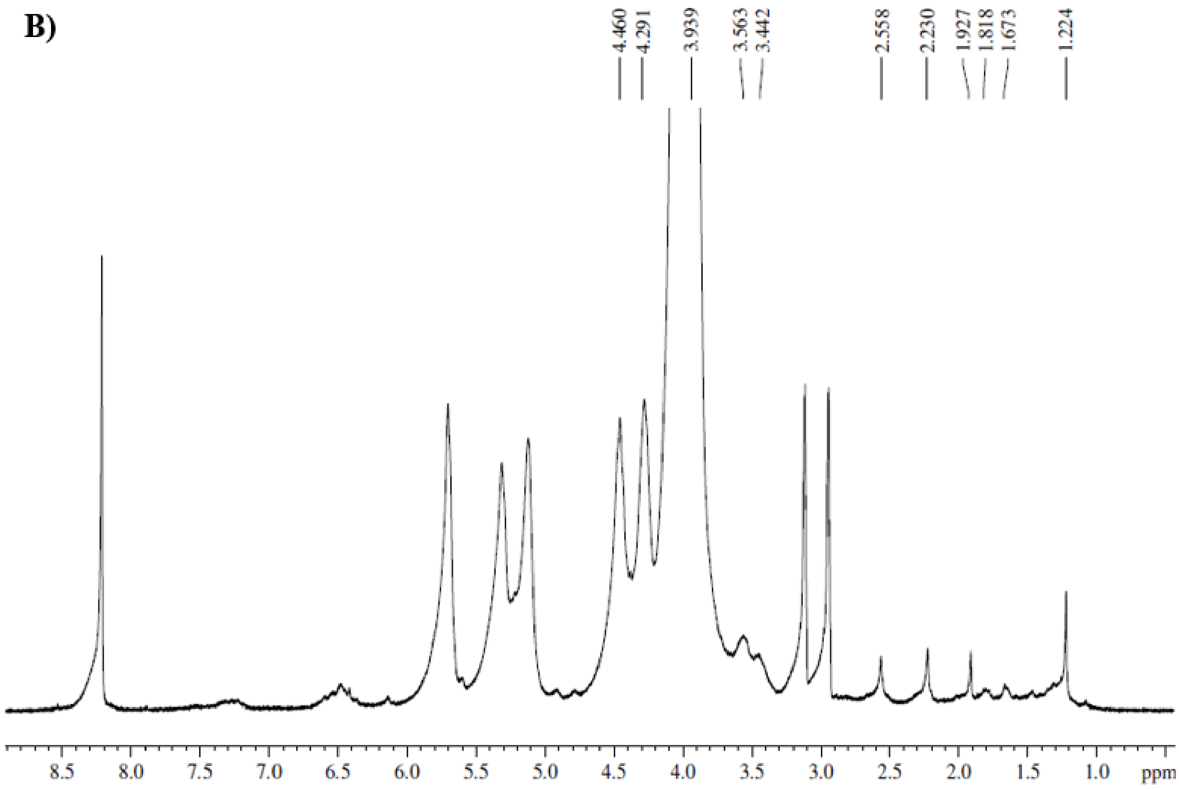

Figure S1. ^{1}H-NMR spectra of A) INU-EDA-TAU and B) INU-EDA-TAU-RA.

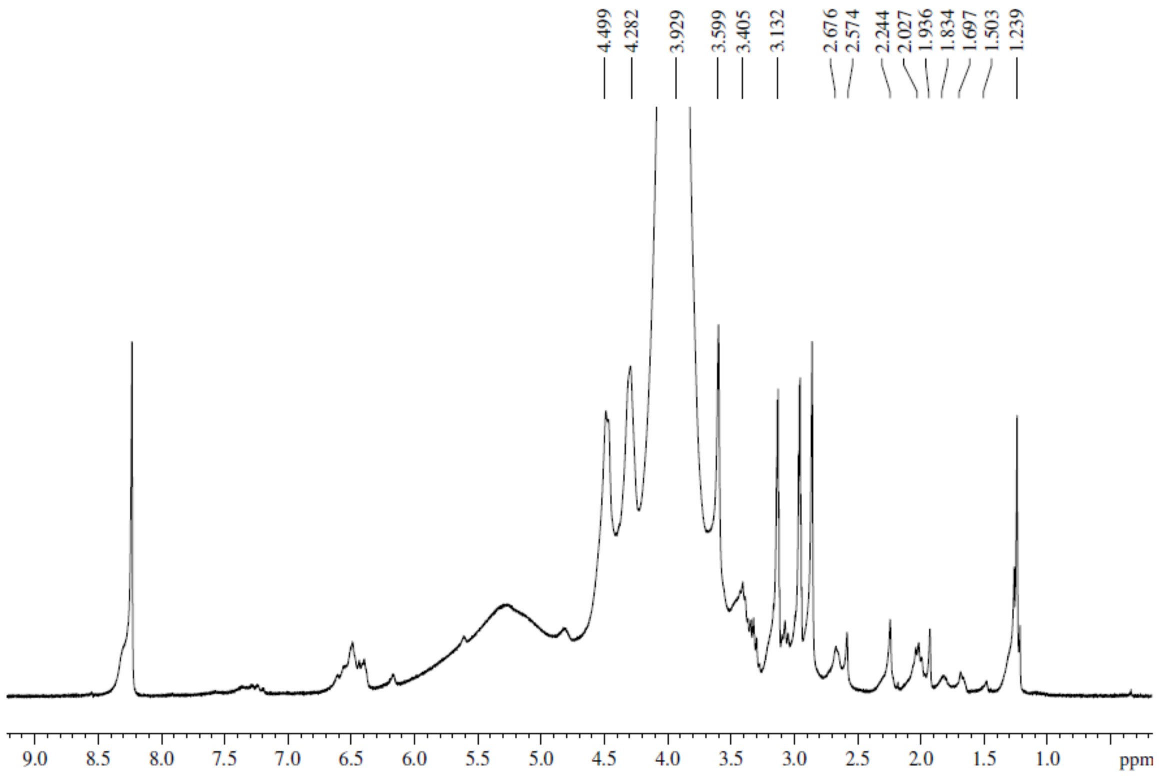

Figure S2. ^{1}H-NMR spectrum of INU-EDA-RA-CAR.

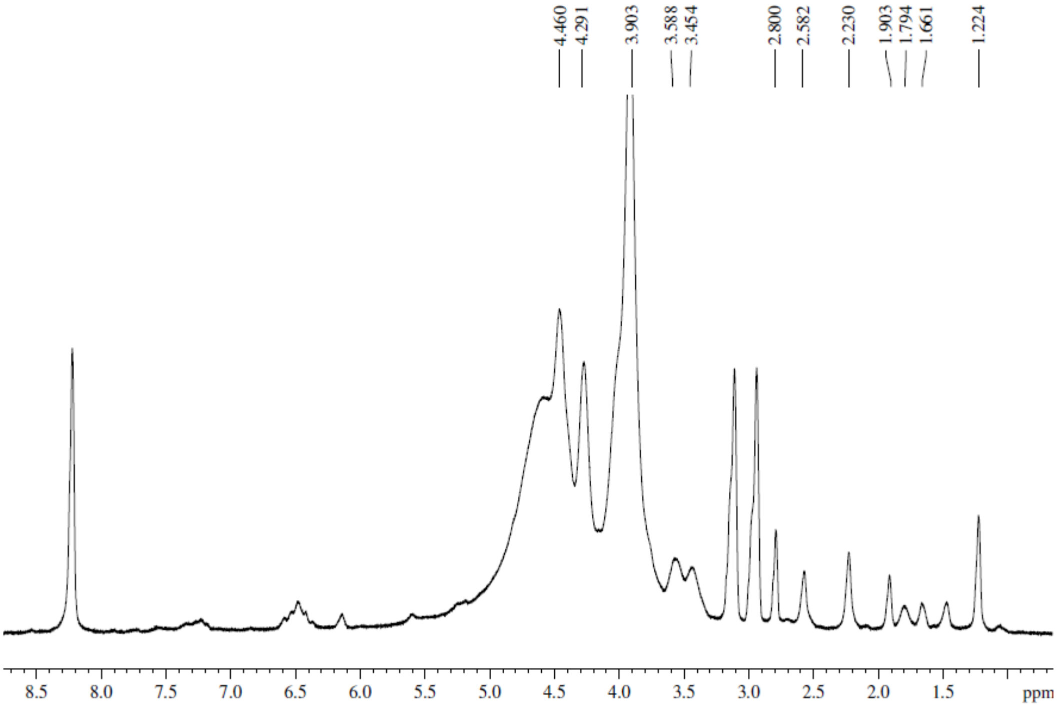

Figure S3. ^{1}H-NMR spectrum of INU-EDA-RA-CRE.

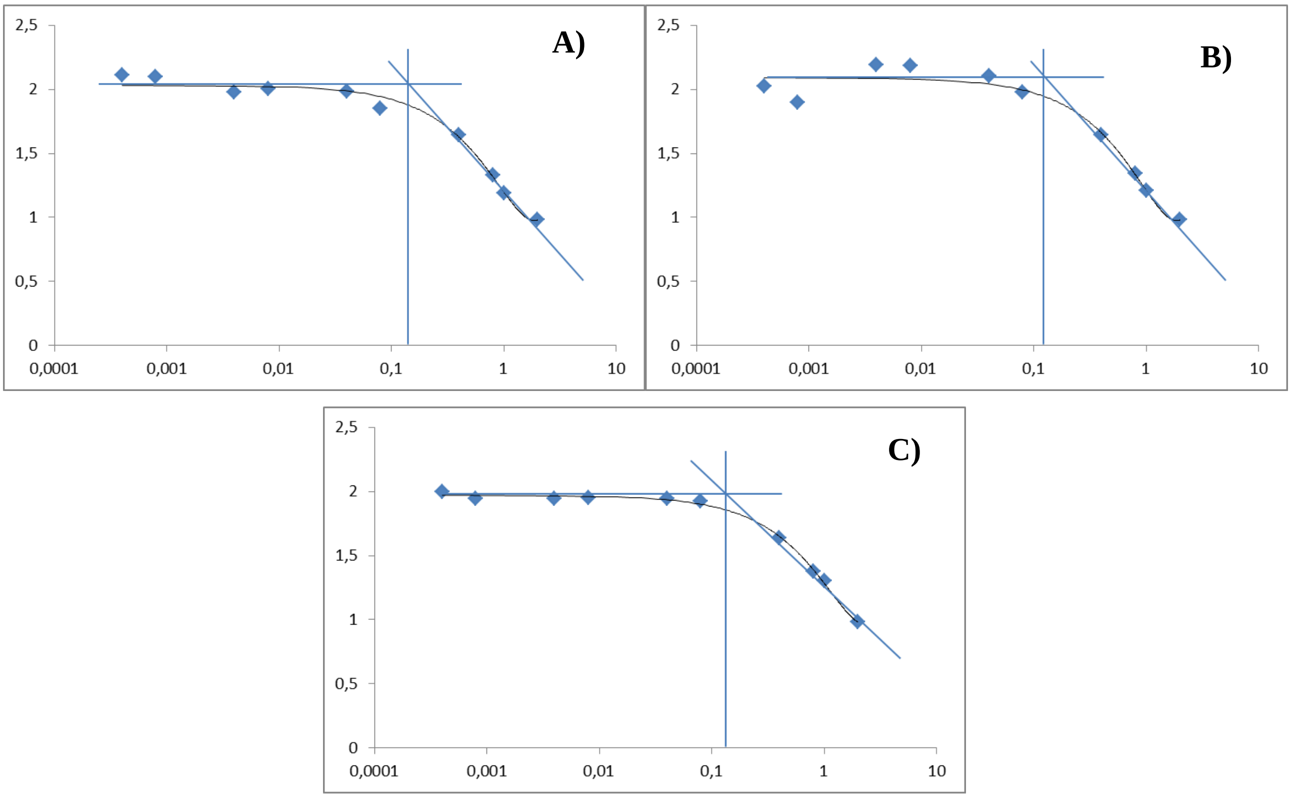

Figure S4. Evaluation of critical aggregation concentration of INU-EDA-TAU-RA micelles in A) bidistilled water, B) DPBS pH 7.4 and C) HEPES pH 7.4: pyrene I_{373}/I_{384} ratio as a function of polymer concentration (log).

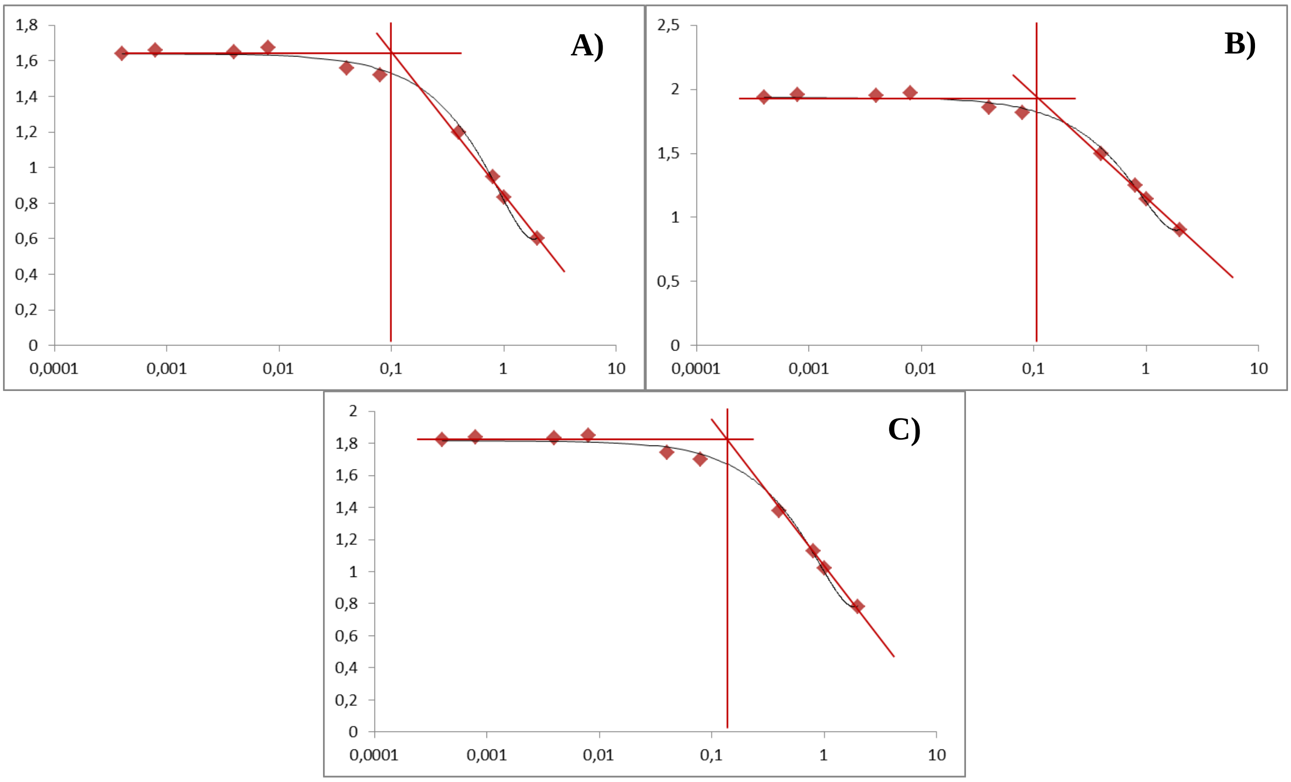

Figure S5. Evaluation of critical aggregation concentration of INU-EDA-RA-CAR micelles in A) bidistilled water, B) DPBS pH 7.4 and C) HEPES pH 7.4: pyrene I_{373}/I_{384} ratio as a function of polymer concentration (log).

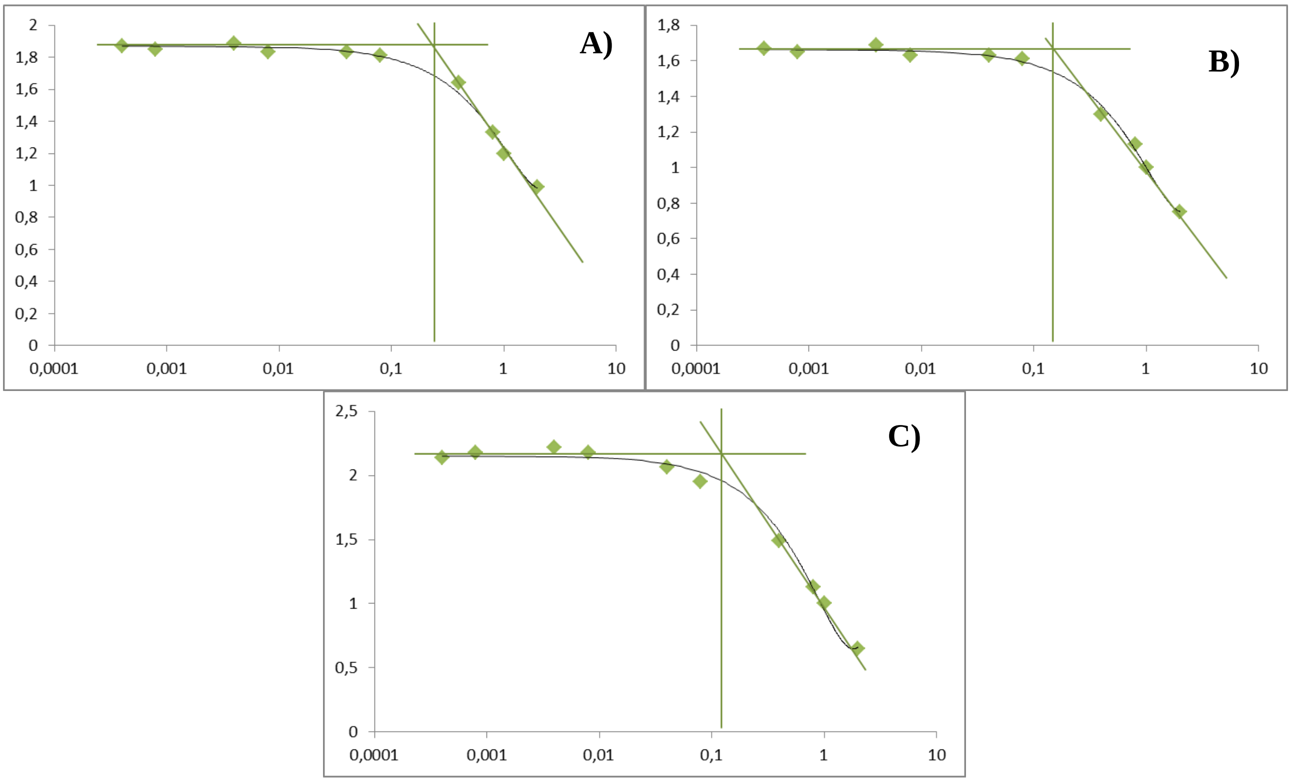

Figure S6. Evaluation of critical aggregation concentration of INU-EDA-RA-CRE micelles in A) bidistilled water, B) DPBS pH 7.4 and C) HEPES pH 7.4: pyrene I_{373}/I_{384} ratio as a function of polymer concentration (log).

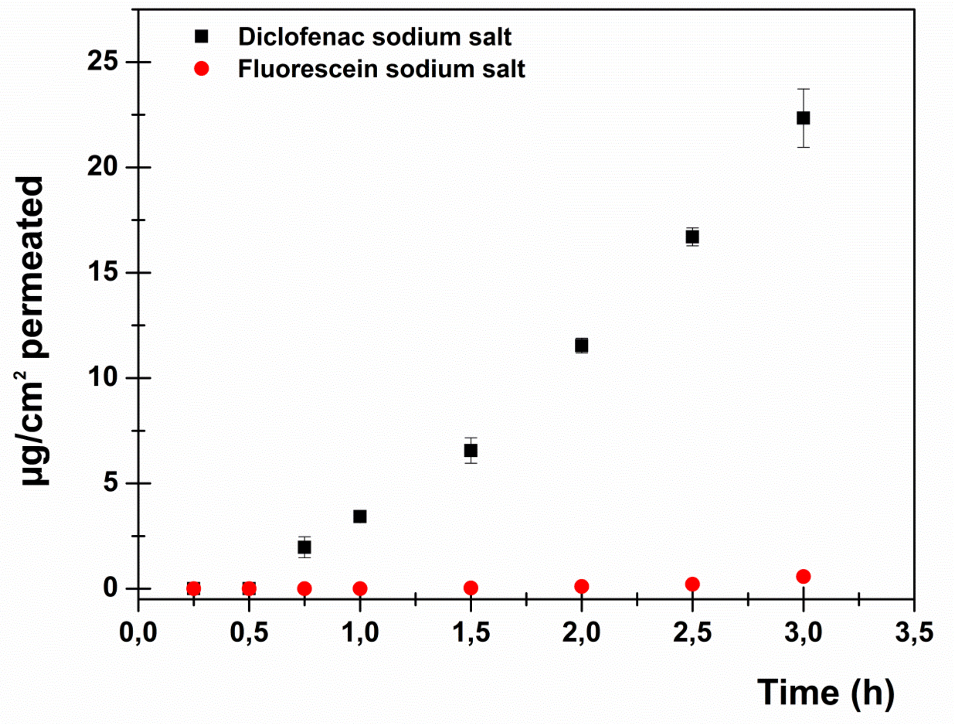

Figure S7. Ex vivo permeation studies: µg/cm² of diclofenac sodium salt (black square) and fluorescein sodium salt (red circle) permeated as a function of incubation time (h).

Table S1. Stability studies performed on freeze-dried DEX-loaded micelles. Particle size (nm), PDI, Z-potential (mV) and DL% ± standard error calculated after sample preparation (time point 0) and after 1, 2, 3 and 6 months of storage at room temperature in the dark.

Sample	Time Point (month)	DL % (w/w)	Particle Size (nm)	PDI	Z-Potential (mV)
INU-EDA-TAU-RA/DEX	0	6.60 ± 0.56	250.63 ± 18.17	0.102	-3.22 ± 0.54
	1	6.52 ± 0.32	245.21 ± 13.22	0.106	-3.00 ± 0.33
	2	6.55 ± 0.49	238.48 ± 20.21	0.122	-2.99 ± 0.59
	3	6.77 ± 0.65	255.20 ± 12.88	0.112	-3.15 ± 0.44
	6	6.53 ± 0.35	228.33 ± 19.31	0.120	-2.98 ± 0.60
INU-EDA-RA-CAR/DEX	0	4.10 ± 0.33	230.60 ± 14.14	0.109	-1.22 ± 0.21
	1	4.00 ± 0.22	228.27 ± 16.28	0.106	-1.50 ± 0.22
	2	4.12 ± 0.41	235.35 ± 12.25	0.110	-1.02 ± 0.13
	3	4.23 ± 0.39	225.58 ± 20.21	0.104	-1.10 ± 0.30
	6	4.12 ± 0.35	226.52 ± 10.43	0.105	-1.00 ± 0.49
INU-EDA-RA-CRE/DEX	0	4.20 ± 0.20	250.60 ± 19.22	0.191	-3.63 ± 0.33
	1	4.21 ± 0.11	234.49 ± 18.01	0.186	-3.20 ± 0.41
	2	4.12 ± 0.30	259.00 ± 22.06	0.157	-3.84 ± 0.26
	3	4.06 ± 0.15	247.65 ± 23.09	0.190	-3.55 ± 0.29
	6	4.23 ± 0.32	252.10 ± 12.55	0.179	-3.94 ± 0.42

Table S2. Kp (cm/s) ± standard error calculated by the ex vivo permeation studies after administration of DICL and FLUO solution and compared with the literature ones (Pescina et al. 2015).

Model Molecule	$Kp_{experimental}$ (cm/s) *	$Kp_{literature}$ (cm/s) **
Diclofenac sodium salt	$(2.9 \pm 0.2)\times10^{-6}$	$(5.3 \pm 0.2)\times10^{-6}$
Fluorescein sodium salt	$(1.3 \pm 0.2)\times10^{-7}$	$(5.0 \pm 2.0)\times10^{-7}$

* bovine corneas; ** porcine corneas
